# Children’s screentime is associated with reduced brain activation during an inhibitory control task: A pilot EEG study

**DOI:** 10.3389/fcogn.2023.1018096

**Published:** 2023-01-22

**Authors:** Kaitlin M. Lewin, Dar Meshi, Fashina Aladé, Erica Lescht, Caryn Herring, Dhatri S. Devaraju, Amanda Hampton Wray

**Affiliations:** 1 Department of Advertising and Public Relations, Michigan State University, East Lansing, MI, United States; 2 Department of Communication Science and Disorders, University of Pittsburgh, Pittsburgh, PA, United States; 3 Department of Communicative Sciences and Disorders, Michigan State University, East Lansing, MI, United States

**Keywords:** screentime, inhibition, Go/No-Go, EEG, ERP, children

## Abstract

Children’s screentime has been linked with a variety of behavioral consequences, including decreased inhibitory control. While children’s screentime is associated with distinct functional brain differences, the links between screentime and neural markers of inhibitory control are unknown. Therefore, we examined these relationships in a pilot study using a Go/No-Go task (*N* = 20). After controlling for age, increased child screentime was significantly correlated with reduced P2 and P3 amplitudes elicited by No-Go trials. No significant relationships were observed with behavioral accuracy or response time. These findings indicate that children with greater screentime exhibit less robust neural processes for inhibitory control. Limitations and future directions are discussed within these preliminary findings.

## Introduction

Screentime refers to the duration of a sedentary activity in front of a screen, such as watching TV, using a computer, or playing video games. Since 1997, children’s screentime has more than doubled (Chen and Adler, 2019; Kaneshiro, 2021), and children aged 9–12 years currently engage with screens for an average of 5 h daily, separate from educational purposes (Rideout and Robb, 2019; Auxier et al., 2020). The most common screen devices used include televisions (88%), tablets (67%), and smartphones (60%), with YouTube being the most dominant platform (Auxier et al., 2020; Thomas et al., 2020). By the age of eight, 19% of children own their own smartphone, rising to 53% by the age of 11 (Rideout and Robb, 2019). Due to these statistics, some parents have reported concern (Auxier et al., 2020), and these worries appear to be supported with initial research. For example, increased screentime in children has been associated with greater risk of delayed language acquisition, decreased IQ, increased aggression, and increased risk for depression and anxiety (Pagani et al., 2011; Byeon and Hong, 2015; Chonchaiya et al., 2015; Maras et al., 2015; Takeuchi et al., 2015). Longitudinally, one study revealed that increased screentime at ages two, three, and 5 years was associated with poorer performance on a task assessing communication, motor skills, problem solving, and social developmental milestones when assessed at each successive follow up evaluation (Madigan et al., 2019).

Children’s screentime has also been investigated with respect to inhibitory control. Inhibitory control is one aspect of executive function (EF), a multidimensional concept related to controlling cognition and behavior, which includes other distinct factors in children such as task switching ability (Lee et al., 2013). Research suggests that both EF overall and inhibitory control increase as children develop (Carlson, 2005). One frequently-used assessment of inhibitory control is a Go/No-Go Task, where participants attempt to withhold a button press for certain stimuli while pressing a button for all other stimuli (Donders, 1969). Researchers have recently used this task to investigate the effects of screentime in preschool-age children and found greater screentime to be associated with lower performance accuracy on this task of inhibitory control. Consistent screentime exposure, based on parental report, also predicted reduced inhibitory control in infants 10 months after screentime assessment (McHarg et al., 2020a), and decreased EF skills on tasks such as the dimensional change card sort and Stroop tasks in toddlers 1 year after screentime assessment (McHarg et al., 2020b). Together, these findings indicate that increased screentime may be associated with reduced inhibitory control in young children.

Researchers have also begun to investigate children’s screentime with neuroimaging methods. In one study, parents reported the amount of time children spent using screens and reading a book. Children then underwent resting-state magnetic resonance imaging to assess connectivity between the left fusiform gyrus [an area associated with visual word formation (Dehaene, 2013)] and brain regions associated with visual, language, and cognitive processing (Horowitz-Kraus and Hutton, 2018). In this study, time spent reading was associated with increased functional connectivity between visual word form area and language and cognitive control areas. In contrast, increased screentime was associated with reduced connectivity with these same regions, specifically those involved in language processing (e.g., angular gyrus) and cognitive control (e.g., insular cortex, inferior frontal gyrus), suggesting consequences of high levels of screentime on language and executive function skills (Horowitz-Kraus and Hutton, 2018). In an electroencephalography (EEG) study, children were exposed to either screen-based or in-person (control group) stories over a 6-week period, and then completed a variety of attention tasks (Zivan et al., 2019). Children in the screen-based story condition demonstrated higher theta vs. beta band activity at rest, a pattern not observed in the control group. Increased theta band activity in children has been associated with poor attentional control (Putman et al., 2010; van Son et al., 2019). These studies provide initial support for a neural mechanism substantiating screentime’s relationship with aspects of executive function in children.

Other neuroimaging studies in children have examined brain function during inhibitory control, primarily through EEG and event-related potentials (ERPs), however without relating these measures to screentime. These studies have focused on the N2 and P3 in reflecting various aspects of inhibitory control. The N2 is believed to index conflict monitoring, or increased engagement when the expected task changes (Donkers and van Boxtel, 2004). For example, when the task is to respond to the majority of stimuli, such as Go trials in a Go/No-Go task, then to refrain from responding to rare, No-Go trials, the task changes for No-Go trials – the task change is to inhibit the expected response, requiring engaged conflict monitoring which is reflected in increased N2 amplitudes. Children display larger N2 amplitudes than adults, with amplitudes decreasing with age for No-Go trials, believed to reflect improved conflict monitoring skills over time (Donkers and van Boxtel, 2004).

In inhibition tasks such as Go/No-Go, P3 is believed to reflect response inhibition with larger amplitudes elicited by No-Go compared to Go trials (Kok, 1986; Donkers and van Boxtel, 2004). P3 amplitudes elicited by No-Go trials increase with age, reflecting improved inhibitory control (Bruin et al., 2001; Jonkman, 2006). Generally, larger N2 and P3 amplitudes elicited by No-Go compared to Go trials reflect increased neural resources needed for successful inhibitory trials. Previous studies employing a Go/No-Go task have found these patterns in children as young as 4 years. Therefore, if N2 and P3 ERP components represent early neural markers of inhibitory control, we may see differences as a function of other factors that influence inhibitory control, such as screentime.

While research has consistently supported the association between inhibitory control and N2 and P3 amplitudes in children, relationships with the N1 and P2 are less documented. N1 amplitudes are sensitive to physical traits of stimuli and are believed to index aspects of attentional control (Näätänen and Picton, 1987; Slagter et al., 2016). Larger N1 amplitudes elicited by No-Go compared to Go trials may reflect increased attention when needing to inhibit a response in both children and adults, though scalp distribution changes slightly with age (Johnstone et al., 2005). P2 is also sensitive to stimulus traits and may index identification and classification of stimuli (García-Larrea et al., 1992; Crowley and Colrain, 2004). In children, larger P2 amplitudes have been elicited for Go compared to No-Go trials, though some adults have exhibited opposite patterns (Lindholm and Koriath, 1985; Ceponiene et al., 2002; Johnstone et al., 2005). Although there is preliminary evidence for the modulation of N1 and P2 during inhibitory control tasks in children, limited research constrains predictions, with differences in inhibitory control and screentime being explored in the current study.

While the neural processes underlying inhibitory control in children are fairly well-understood (Lindholm and Koriath, 1985; Ceponiene et al., 2002; Johnstone et al., 2005, 2007; Jonkman, 2006; Cragg et al., 2009; Abdul Rahman et al., 2017; Elke and Wiebe, 2017), the relationships between these processes and screentime are unknown. Although relationships between behavioral responses and screentime have been reported, the extant literature spans across multiple different development stages and age ranges. However, reports indicate that relationships with screentime use are of specific concern across all ages for children under 12 years (Auxier et al., 2020). Furthermore, characterizing relationships between neural processes for inhibitory control and screentime provides greater specificity as to which aspects of inhibitory control – e.g., attention, conflict monitoring, response inhibition – may differ as a function of screentime in children. This study takes a first step toward addressing this gap; we evaluated relationships between children’s daily screentime and behavioral and neural markers of inhibitory control in a pilot study of 20 children. Parents provided daily reports of children’s screentime for two consecutive weeks to generate an average screentime measure, and children completed a visual Go/No-Go task, during which behavioral performance and ERPs were acquired. With the above research in mind, we hypothesized that greater screentime in children would be associated with decreased task performance (i.e., reduced accuracy and slower response times), as well as reduced N2 and P3 amplitudes elicited by No-Go trials. We also explored differences in N1 and P2 components, without specific hypotheses due to mixed findings in children.

## Method

### Participants

Participants included 23 children, ages 3.8–10.1 years. All children were recruited as part of a larger study of attentional control in developmental stuttering. Only children with no history of stuttering, and who did not stutter at the time of assessment, were included. Inclusion criteria included being a monolingual speaker of English, no history of neurological disease or injury, no history of speech, language, or hearing disorder, completion of all daily screentime questionnaires (see below), and completion of the Go/No-Go EEG paradigm. One child was excluded for excessive artifacts in their EEG data, rendering the data unusable, and two participants were excluded due to low response accuracy on the Go/No-Go tasks, more than 2.5 SD below the mean. The final group of 20 children (M = 6.26 years, *SD* = *1.5*) included 13 males and 7 females. All but one child was in school (four preschool, eight kindergarten, two 1^st^ grade, two 2^nd^ grade, two 3^rd^ grade, one 5^th^ grade, one stayed home [age 3.8 years]). Data regarding the highest level of parental education were collected and coded as years of education completed, adapted from a consensus measure (Pollak and Wolfe, 2020): 10 = some high school, 12 = completed high school, 13 = partial college, 14 = completed 2-year degree, 16 = standard college/Bachelor’s degree, 18 = graduate school or professional degree. Parents also provided annual household income level using an incremental scale from $0–$10,000 to over $250,000. Overall, participant families had an average parental education level of a 4-year college degree and were in the middle income level in the United States [Kochhar, 2018; see Table 1]. Parents reported 17 children as White, 1 as Black, and 2 as more than one race. All children passed a hearing screening at 20 dB HL at 500, 1 K, 2 K, and 4 KHz and had normal or corrected-to-normal vision. Study procedures were approved by the Institutional Review Board at Michigan State University.

### Behavioral measures

All children completed a battery of behavioral assessments to evaluate language and nonverbal intelligence abilities. Language skills were assessed using the Clinical Evaluations of Language Fundamentals, Fifth Ed [CELF-5; Wiig et al., 2013] or Preschool, Second Ed [CELF-P2; Wiig et al., 2004], depending on participant age (CELF-5 for children >5 years). The Primary Test of Non-verbal Intelligence [PTONI; Ehrler and McGhee, 2008] was administered to assess non-verbal intelligence. One child, age 10.1 years, completed the Test of Non-verbal Intelligence, Fourth Edition [TONI; Brown et al., 2010] as their age exceeded the standardized range for PTONI. All children performed within the normal range on both assessments. Demographic characteristics and descriptive statistics of survey data are presented in Table 1.

### Daily screentime measure

As part of participant case history report, prior to knowing about the screentime questionnaire, parents provided an estimate of the average amount of time their child spent watching TV and playing video games per week, consistent with previous studies of screentime.

To provide a more detailed measure of screentime, parents provided information about screentime once per day for two consecutive weeks. One parent of each participating child received a daily text at 9 pm that contained the link to an online survey (see Appendix). All parents responded to the questions regarding their child’s daily screentime for 14 days. The first question asked whether the child spent time on any electronic device *outside of school hours*. Device options included Computer, Smartphone or Tablet, E-reader, Television, Video Game Device, Other Device, or No Screentime. If they responded that the child had used any of the aforementioned devices that day, parents were then asked to estimate the duration of time their child spent on each selected device with drop-down options to provide information in Hours (0–23) and Minutes (0–60)^1^. We calculated average daily screentime by summing time spent on each device for all 14 days, then dividing by the total number of days.

### Go/No-Go task

**FIGURE 1.**
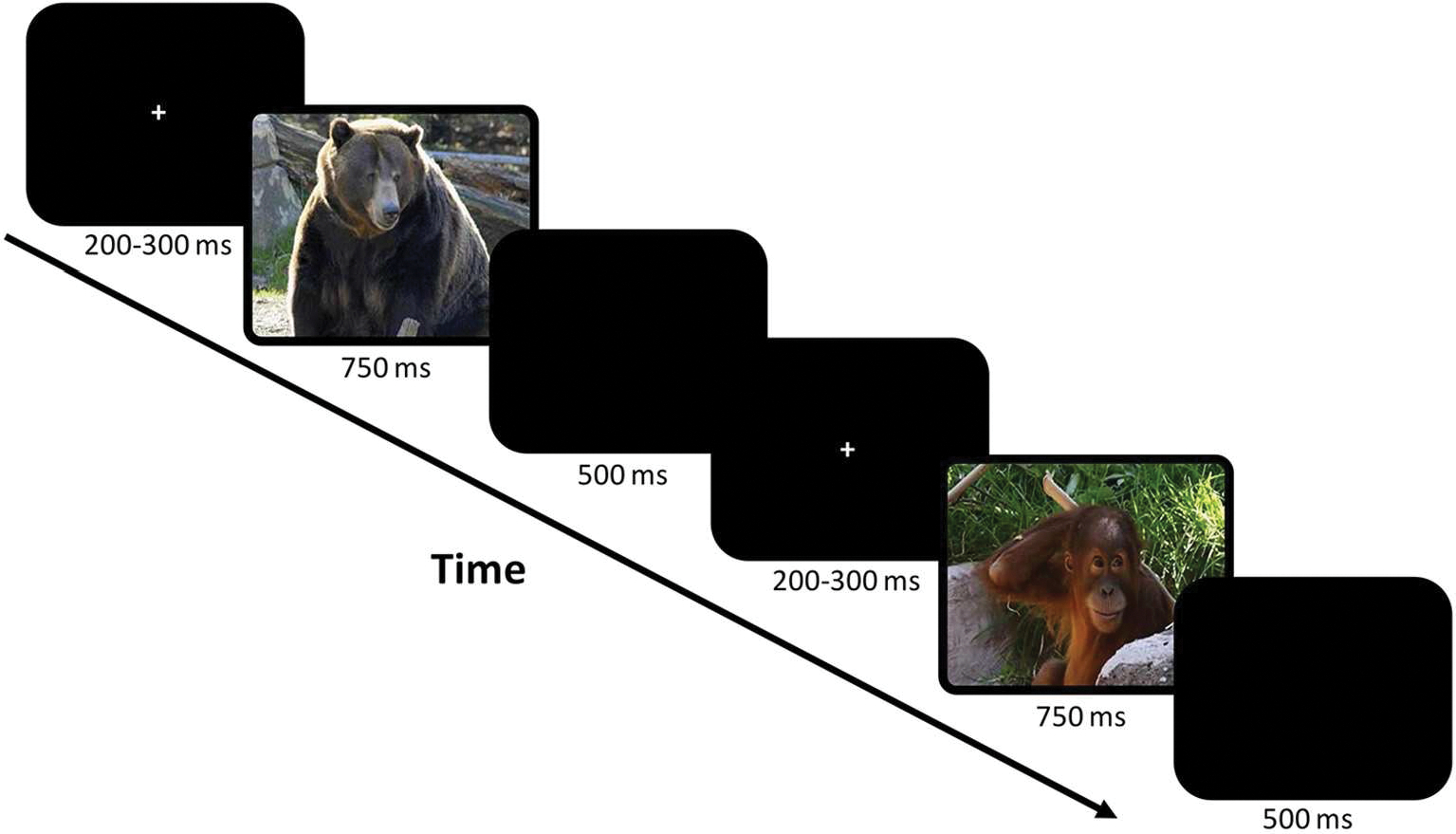
The child-friendly visual Go/No-Go zoo task (Grammer et al., 2014). This task uses pictures of animals as stimuli in a game format to keep children engaged. Children were instructed to press a button as quickly as possible when they saw an animal (Go trials) except for orangutans (No-Go trials). Example of two trials including the fixation and a blank screen are shown in the figure.

Electrophysiological data were acquired while children completed a child-friendly visual Go/No-Go Zoo paradigm (Grammer et al., 2014). Children were presented with a narrative; the animals in the zoo had escaped from their cages. Their job was to help the zookeeper return the animals to their cages by pressing a designated button as quickly as possible when they saw any animal (Go trials) *except* the orangutans (No-Go trials), who were helping the zookeeper. All stimuli were presented on a monitor directly in front of the participant at a distance of 142 cm *via* ePrime (Grammer et al., 2014). Children completed a training block (9 Go and 3 No-Go trials) to ensure they understood the task. Then children completed 8 testing blocks, each consisting of 30 Go and 10 No-Go trials. Each trial began with a fixation cross jittered for 200–300 ms, then the stimulus/animal picture (either Go or No-Go trial) was presented for 750 ms (Figure 1). A blank screen was then presented for 500 ms. Responses *via* button press on a response pad with the right hand were recorded while the stimulus picture was presented or during the sub-sequent 500 ms period. Go and No-Go trials were interspersed throughout each block and stimuli were balanced between and within blocks for animal type, color, and size. Behavioral accuracy was calculated separately for Go and No-Go trials, and response times were calculated only for correct Go trials (as “no response” was correct for No-Go trials).

### Procedure

Children arrived at the lab and were introduced to the EEG equipment and tasks. Parents provided signed consent for participants and all children provided verbal assent as well as signed/written child assent if above the age of seven. Children completed the battery of behavioral measures and the EEG task in two separate sessions on separate days, typically within a 30-day period. For EEG testing, an electrode cap was placed on the child and they were seated in a quiet, sound-attenuating booth with a trained research assistant. Children then received instructions for the Go/No-Go task and completed the training and testing blocks. Upon completion of the EEG testing session, if parents agreed to participate in the daily screentime survey, they received a follow-up email with instructions and began receiving daily text questionnaires.

### Electrophysiological data acquisition

EEG data were acquired *via* 32 Ag/AgCl electrodes embedded in an elastic cap (Biosemi Active 2, Amsterdam, Netherlands). Electrode locations were consistent with the International 10–20 System. Additional electrodes were placed over the left and right mastoids as well as the left and right outer canthi and left orbital ridge to monitor eye movements. EEG data were recorded unreferenced and unfiltered and relative to the common mode sense electrode, as is standard for Biosemi data collection, at 512 Hz. Electrode offsets, similar to impedance measures, were less than ±20 mV for all electrodes for all participants, less than the recommended ±40 mV (Biosemi).

EEG data were processed using the EEGLAB (Delorme and Makeig, 2004) in MATLAB (The MathWorks Inc., Natick, MA, USA). Offiine, data were re-referenced to the average of the left and right mastoids and down sampled to 128 Hz. Data were high-pass filtered at 0.01 Hz using a hamming window finite impulse response (FIR) filter with an order of 846 and were low-pass filtered at 30 Hz using a hamming window FIR filter with an order of 424. Artifact subspace reconstruction as implemented in EEGLAB (Delorme and Makeig, 2004; Mullen et al., 2015) was used to remove burst and drift artifacts, and bad channels were interpolated. Independent component analysis (ICA) was performed and components containing ocular artifacts were removed based on the topography scalp map, component time course and power spectrum. EEG data were epoched between 200 ms prior to and 1,200 ms after the onset of each stimulus, with the 200 ms prior to stimulus onset used for baseline correction. Epochs containing artifact that exceeded ±200 mV in any channel across the epoch window were automatically rejected. For each child, remaining epochs were averaged separately for Go and No-Go trials. The mean (*SD*) number of trials included was 234.8 (*9.8*), 97.8%, for Go trials and 77.8 (*4.0*), 97.2%, for No-Go trials.

### Data analysis

#### Electrophysiological data analysis

**FIGURE 2.**
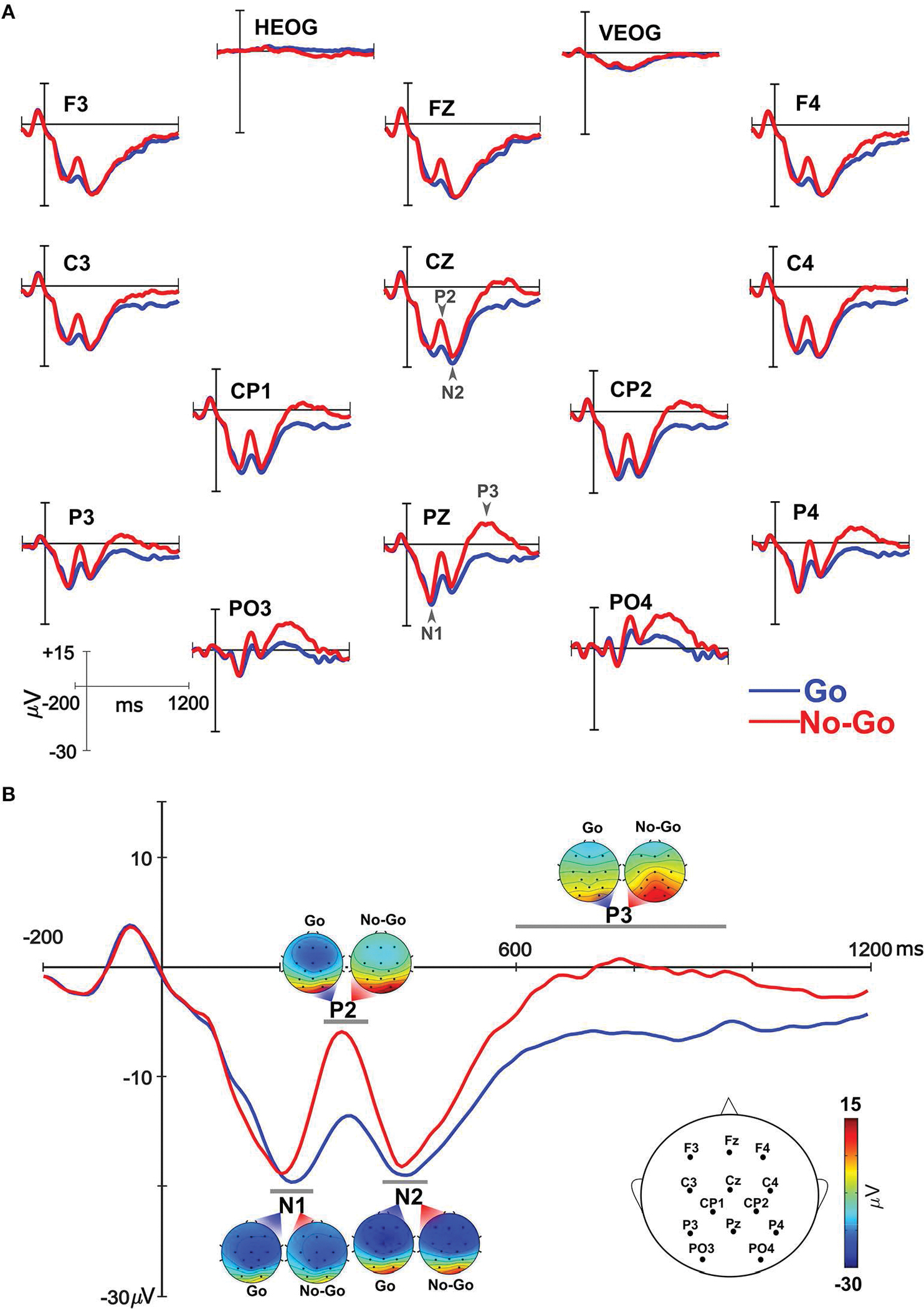
Electrophysiological responses elicited by Go (blue) and No-Go (red) trials. **(A)** Event-related brain potentials (ERPs) over all the electrode locations included in the analysis. Each component – N1, P2, N2, P3 – is marked with an arrow. **(B)** Composite ERP waveform from all electrode locations included in the analyses (see head insert specific for electrodes included). Scalp topography of each component for Go and No-Go trials are illustrated.

ERPs elicited by the Go and No-Go stimuli included N1, P2, N2, and P3 (Figure 2). Given the narrow peaks for N1, P2, and N2, ERP mean amplitudes were extracted across a 60 ms time window centered on the peak of the component (e.g., peak = 230 ms, mean amplitude time window = 200–260 ms). The peak for each component was determined from where it occurred in a grand averaged waveform (Supplementary Figure 1). Consistent with previous studies, aggregate electrode sites, or regions of interest (ROIs), were created for each component for each condition for each participant. ROIs were selected based on previous Go/No-Go studies (Kiefer et al., 1998; Jonkman et al., 2003; Jonkman, 2006; Lamm et al., 2012) and included electrodes distributed across the scalp for N1, P2, and N2, and with a centroparietal distribution for P3 (see Supplementary Figure 2). These locations align with the most prominent distribution of each component in the current dataset. Electrode locations included in analyses are illustrated in Figure 2. ROIs were created by averaging the mean amplitudes across the specified set of electrodes. For N1, mean amplitudes were calculated between 200–260 ms and averaged across F3/4, C3/4, CP1/2, P3/4, FZ, CZ, and PZ. P2 mean amplitudes were calculated between 280–340 ms and averaged across F3/4, C3/4, CP1/2, P3/4, PO3/4, FZ, CZ, and PZ. Mean amplitudes for N2 were calculated between 390–450 ms and averaged across F3/4, C3/4, CP1/2, P3/4, FZ, CZ, and PZ. The P3 component had a broader time scale and centroparietal distribution, therefore mean amplitudes were calculated across a wider time window still centered on the peak, 600–950 ms, and averaged across C3/4, CP1/2, P3/4, PO3/4, CZ, and PZ. ERP data were analyzed using a repeated-measures ANCOVA with within-subject factors of condition (Go, No-Go) and a covariate of age. Alpha was set at 0.05 and partial eta squared values are reported for all significant effects.

#### Data analysis to relate screentime, task performance, and ERPs

All statistical analyses involving survey and behavioral data were performed using SPSS (version 27), except confidence intervals for first-order correlations, which were calculated in R Version 4.0 (R Core Team, 2019) utilizing the “Rmimic” R package [version 1.0.3; Pontifex, 2020]. Paired sample *t*-tests were conducted to determine behavioral performance differences between Go and No-Go trial accuracy, and differences between parents’ preliminary self-reported child screentime and our 14-day screentime measure. To investigate the relationships between average screentime and the four ERP component mean amplitudes elicited during both Go and No-Go trials, we conducted first-order partial Pearson’s correlations, controlling for age. We also conducted first-order partial Pearson’s correlations between screentime and behavioral performance (accuracy, response time), controlling for age. For all *t*-tests and first-order correlations, alpha was set at 0.05.

## Results

### Survey and task behavior

Based on the 14-day parental survey of children’s screentime, mean screentime across all participants was 109.02 min per day (*SD* = *64.3,* Table 1). Although parent estimates of screentime were lower than using a daily measure, the 2-week measure of screentime did not different statistically from parents’ preliminary child screentime estimate of 87.43 min per day [*SD* = *57.02*, *t*_(19)_ = 1.45, *p* > 0.05]. Mean accuracy and response times for the Go/No-Go task are presented in Table 1. Participants correctly responded to 95.3% of Go trials and 59.8% of No-Go trials. Accuracy differed between conditions, with children performing with greater accuracy on Go compared to No-Go trials [t_(19)_ = 7.06, *p* < 0.001]. Average response time for participants on Go Trials was 411.7 ms (SD = *76.9*). First-order partial correlations controlling for age revealed no significant relationships between screentime and Go accuracy, No-Go accuracy, or Go response time (all *p’*s > 0.05).

### ERPs

Grand average ERP waveforms elicited by the Go and No-Go stimuli are illustrated in Figure 2. Scalp distribution for each component within the analysis time window illustrate frontocentral distribution of N1 and N2 and a centroparietal distribution for P2 and P3 (Figure 2B). Visual inspection of the data suggested larger N1 mean amplitudes elicited by Go compared to No-Go trials while No-Go trials elicited larger P2 and P3 mean amplitudes. Statistical analyses confirmed these patterns. N1 mean amplitudes elicited by Go trials were larger than those elicited by No-Go trials [F_(1,18)_ = 4.90, *p* = 0.040, $n_{p}^{2}\;=\;0.21$]. For P2, mean amplitudes elicited by No-Go trials were larger than those for Go trials [F_(1,18)_ = 30.59, *p* < 0.001, $n_{p}^{2}\;=\;0.63$]. P3 mean amplitudes were also larger for No-Go compared to Go trials [F_(1,18)_ = 7.10, *p* = 0.016, $n_{p}^{2}=0.28$]. No differences were observed in N2 mean amplitudes between Go and No-Go trials [F_(1,18)_ = 3.71, *p* = 0.07].

**FIGURE 3.**
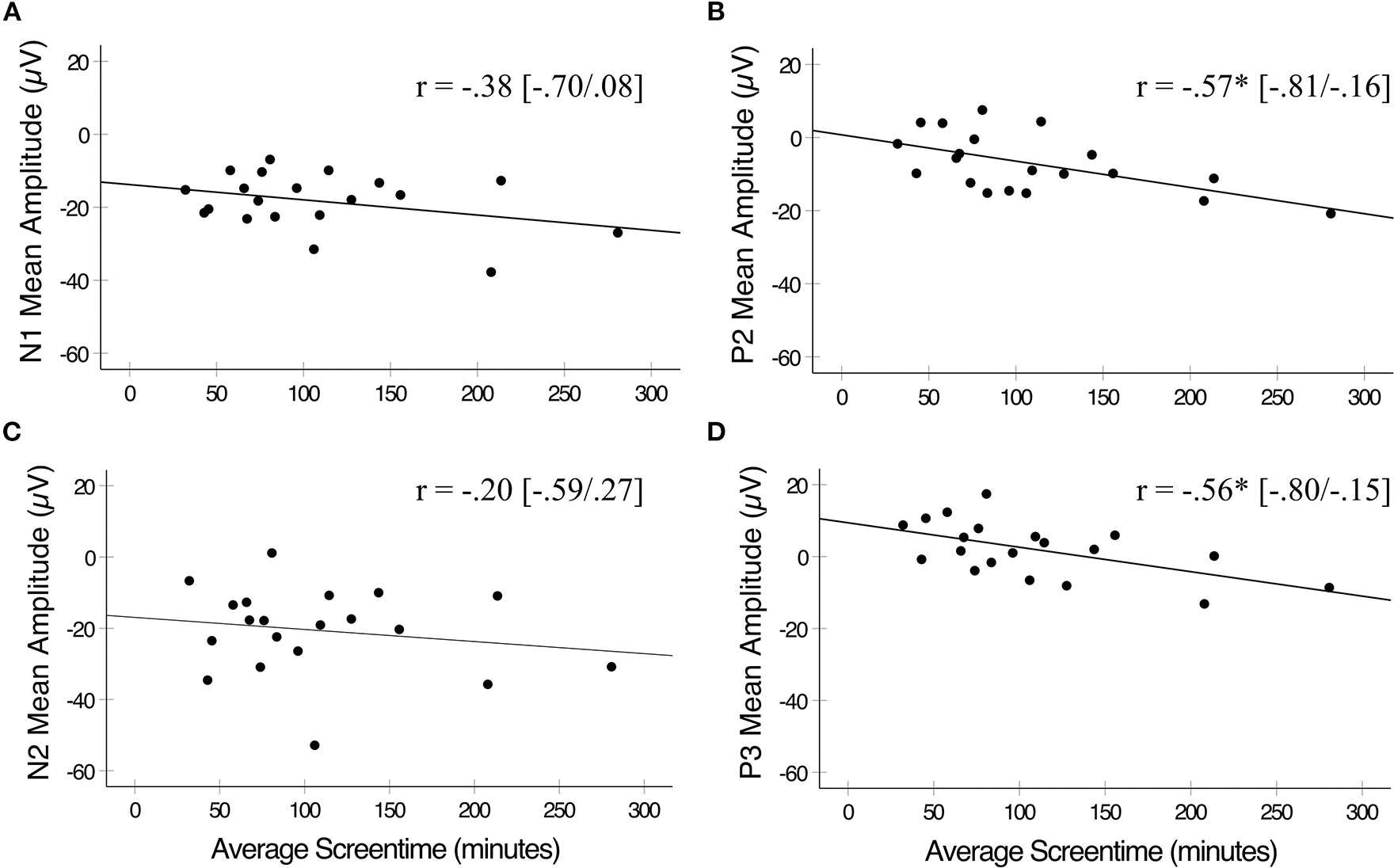
Pearson’s correlations between average daily screentime and ERP mean amplitudes elicited by No-Go trials. **(A)** Average screentime was not significantly correlated with N1 mean amplitudes. **(B)** Average daily screentime was inversely correlated with P2 mean amplitudes. **(C)** Average daily screentime was not significantly correlated with N2 mean amplitudes. **(D)** Average screentime was inversely correlated with P3 mean amplitudes. *p < 0.05. Correlations plotted between X and Y for illustration purposes only. Statistical analyses completed on first-order correlations.

To assess relationships between screentime and ERP components, first-order partial correlations were conducted between average screentime and each ERP measure for No-Go trials, controlling for age (Figure 3). Greater average daily screentime was associated with reduced mean amplitudes for P2 (r = −0.57, 95% C.I. = −0.81, −0.16, *p* = 0.012) and P3 (r = −0.56, 95% C.I. = −0.80, −0.15, *p* = 0.013) elicited by No-Go trials across frontocentral and centroparietal electrodes. Due to the smaller sample size, we conducted post hoc power analyses using G*Power version 3.1 (Faul et al., 2009) for significant correlations, with a sample size of 20 and significance set at α = 0.05. Correlations between average screentime and P2 amplitudes indicated power of 0.79 with a medium effect size of 0.57. Correlations between average screentime and P3 amplitudes indicated power of 0.76 with a medium effect size of 0.56. Therefore, despite the small sample size our significant results still yield adequate power. No significant correlations were observed between a child’s average daily screentime and N1 or N2 mean amplitudes for No-Go trials (r’s < |0.38|, *p’s* > 0.05), and there were no significant correlations between a child’s average daily screentime and any ERP measure for Go trials (*p’s* > 0.05). To correct for multiple comparisons, we divided our α = 0.05 threshold by the four statistical tests of our ERPs of interest and average screentime for a corrected threshold of *p* = 0.013. Therefore, our significant P2 and P3 ERP × screentime correlations analyses survived Bonferroni correction for multiple comparisons.

## Discussion

The current pilot study consisted of a preliminary exploration of how screentime relates to behavioral and neural markers of inhibitory control in children. We hypothesized that increased screentime in children would be associated with reduced inhibitory control (i.e., reduced accuracy and slower response times) and reduced amplitudes of the N2 and P3 ERP components, which have previously been linked with inhibitory control (Jonkman, 2006; Cragg et al., 2009). In addition, we explored relationships between screentime and N1 and P2 amplitudes due to prior mixed findings in their role with inhibitory control in children (Manzi et al., 2011; Elke and Wiebe, 2017). Overall, parents reported almost 2 h of daily screentime per child. While our 14-day daily screentime measure was not significantly different from parent’s preliminary one-time estimate, it was qualitatively higher. As the children within the current study reported lower screentime than the national average (Rideout and Robb, 2019; Auxier et al., 2020; Thomas et al., 2020), these differences may be important in a different group of children who use screens more often. Therefore, daily reporting may improve upon prior literature which typically uses one-time assessments and capture a more accurate indicator of daily screentime patterns and any subsequent relationships with ERP’s. We found no significant relationships between behavioral performance on the Go/No-Go task and screentime. However, increased screentime in children was significantly related to reduced P2 and P3 amplitudes elicited by No-Go trials. While previous research has cited the N2 as a marker of inhibitory control during a Go/No-Go task (Donkers and van Boxtel, 2004; Jonkman, 2006; Cragg et al., 2009), we found no differences in amplitude for N1 or N2 in relation to screentime. In addition, there were no significant differences between screentime and any ERP measure for Go trials.

We found significant differences in accuracy between Go and No-Go trials, with children being less accurate on No-Go trials. However, these differences were not related to screentime, and we did not find any significant associations between behavioral performance on this task and screentime. This is in contrast to previous studies which cited a negative relationship between screentime and behavioral performance on tasks of inhibitory control and broader EF (Martins et al., 2020; McHarg et al., 2020a,b). However, one of the aforementioned studies collapsed EF into a singular category, therefore it is hard to determine the behavioral response in that study associated with specifically inhibitory control (McHarg et al., 2020b). Age could be another factor contributing to the disparity, as previous studies have been carried out in more narrow developmental ranges, compared to the wider age range of the present sample. For example, previous studies in infants and toddlers suggest that very young children (4 mo–5 yrs) display negative relationships between screentime and behavioral inhibition (McHarg et al., 2020a,b). Conversely, a recent study in school-aged children (9–10 yrs) used a stop signal task (another measure of inhibitory control) to reveal a positive relationship between screentime and behavioral inhibition (Chaarani et al., 2022). In this study, children who played video games for a substantial amount of time (21+ h/week) exhibited slightly faster responses (~8 ms) compared to peers who did not play video games (0 h/week), suggesting that high levels of video-game-related screentime may enhance specific aspects of cognition, such as speed of inhibitory control/response times (Manzi et al., 2011). Therefore, our study’s lack of an observed relationship between screentime and behavioral inhibition may be explained by our sample’s broad age range (3.8–10.1 yrs), which spans from toddlers to school-aged children, including both of these periods of development that display directionally opposite relationships between screentime and behavioral inhibition. Future longitudinal studies are needed to provide a more comprehensive characterization of the dynamic relationships between screentime and inhibitory control across development.

In addition, as we utilized an adapted Go/No-Go task for children which involved a story about escaped zoo animals, it could be that performance on this specific version is not related to screentime. Research highlights neural activation differences between various Go/No-Go versions that differ in stimulus timing (Rubia et al., 2001), therefore future research should explore whether other variations within this paradigm, such as visual stimuli or storyline differences, also impact results. Finally, neural markers can precede eventual behavioral change (O’Connell et al., 2009); therefore, future research should investigate how the response timing may influence these relationships.

The present pilot study found that increased screentime in children was significantly linked with reduced No-Go P3 amplitudes. P3 amplitudes elicited by No-Go stimuli are typically largest over centro-parietal and parietal electrode locations and are thought to reflect updating of working memory or inhibition of an ongoing process, such as responding to a stream of Go stimuli (Fonken et al., 2020). Previous studies employing similar tasks in children have established that P3 amplitudes elicited by No-Go trials reflect inhibitory control; specifically, smaller P3 amplitudes have been linked with reduced inhibitory control (Bruin et al., 2001; Johnstone et al., 2005; Jonkman, 2006; Cragg et al., 2009). Of note, this relationship is more prominent in older children, supporting age-related changes in P3 amplitudes, and an overall increase in P3 amplitudes with age (Johnstone et al., 2005; Jonkman, 2006). After controlling for age, our study found that increased screentime in children was linked with *reduced* P3 amplitudes. If reduced P3 amplitudes reflect decreased inhibitory control and increased screentime also relates to reduced P3 amplitudes, together with previous results, our findings suggest that increased screentime may be related to reduced inhibitory control in children.

The current pilot study found that greater screentime in children is related to reduced P2 amplitudes elicited by No-Go trials. Previous studies of relationships between P2 and inhibitory control have yielded inconsistent results. In line with our finding, poorer cognitive control in a task-switching paradigm seems to be linked with reduced P2 amplitudes (Elke and Wiebe, 2017). Similar to the P3, P2 amplitudes elicited by No-Go conditions appear to increase as children age, supporting the belief that increased P2 amplitudes reflect better cognitive control abilities (Elke and Wiebe, 2017). Previous studies have also found reduced P2 amplitudes elicited by a Go/No-Go task in children with ADHD compared to children without ADHD (Smith et al., 2004; Johnstone et al., 2009), further supporting that P2 reflects the inhibition of competing or irrelevant stimuli (Oades, 1998), and reduced P2 amplitudes to reflect decreased cognitive control (Elke and Wiebe, 2017). Thus, our findings of reduced P2 amplitudes in children with increased screentime may suggest reduced or less efficient neural processes for inhibitory control in children with greater daily screentime. If larger P2 and P3 amplitudes during inhibition task trials reflect increased cognitive control (Elke and Wiebe, 2017), then reduced P2 and P3 amplitudes would seemingly reflect the opposite, or decreased inhibitory control. Together, our P2 and P3 findings demonstrate that increased screentime is associated with neural patterns indicative of reduced inhibitory control in children.

To note, in the current Go/No-Go paradigm, larger P2 amplitudes were elicited by No-Go compared to Go stimuli over parietal electrodes sites. Although in many Go/No-Go tasks, larger P2 amplitudes are elicited by Go than No-Go stimuli, the current task has a high degree of variability in Go trials (each Go stimulus is a unique image) and lower variability in No-Go trials (a set of three images are repeated throughout the task). We speculate that the larger P2 amplitudes we observed for the No-Go compared to Go conditions reflect the higher repetition of No-Go stimuli. In other words, the repeated No-Go stimuli allowed for more automatic identification and classification in comparison to the unique Go stimuli (Johnstone et al., 1996, 2005).

While our findings reveal reduced neural processes for inhibitory control in children with greater screentime, there are several limitations to the current study. First and foremost, this is a pilot study with 20 participants of a relatively broad age range (3.8–10.1 years). Although *post-hoc* power analyses suggest the study is adequately powered to support the current findings, results should be interpreted with these sample characteristics and the preliminary nature of our study in mind. Second, although we assessed children’s average daily screentime across a 2-week period, our research still employed a cross-sectional design. Therefore, we cannot address a causal or developmental component beyond the reported relationships. Future research should assess longitudinal neural processes for inhibitory control in relation to children’s screentime, similar to the behavioral inhibitory control and broader EF longitudinal research (McHarg et al., 2020a,b). Third, although we assessed child screentime daily across 14 days, we still relied on parental self-report, which may have biased our findings due to the potential for parents to under-report the frequency of screen use in their children. Future studies may benefit from comparing parental report to actual screentime metrics on devices themselves.

In sum, we found initial support that increased screentime is related to less robust neural processes for inhibitory control, as indicated by reduced P2 and P3 amplitudes in children. However, we did not find associations between screentime and behavioral performance on the Go/No-Go task. Our results are novel as our pilot study is the first to investigate how screentime in children relates to neural markers of inhibitory control. As research has reported children’s screentime usage is increasing in frequency and prevalence (Chen and Adler, 2019), it is important to assess potential detrimental effects on brain and behavioral functions. Our results suggest that these increased screentime frequencies have links with neural markers for reduced inhibitory control in children. Therefore, it may be beneficial for healthcare providers and parents to keep in mind how much their children engage with screens.

## Supplementary Material

Lewin et al 2023 - Supplementary Figure 2 **SUPPLEMENTARY FIGURE 2.** Scalp topographies for each event-related brain potential (ERP) component averaged across the mean amplitude time window for Go (left) and No-Go (right) conditions.

Lewin et al 2023 - Supplementary Figure 1 **SUPPLEMENTARY FIGURE 1.** Grand average event-related brain potential (ERP) waveforms for Go (blue) and No-Go (red) conditions averaged across the electrodes included in the region of interest (bottom right corner) for each component, N1 (top), N2 (top), P2 (middle) and P3 (bottom). Time windows in which mean amplitudes were extracted are shaded in gray.

Supplementary material

The Supplementary Material for this article can be found online at: https://www.frontiersin.org/articles/10.3389/fcogn.2023.1018096/full#supplementary-material

## Figures and Tables

**TABLE 1:** Demographic characteristics, descriptive statistics of survey measures, Go/No-Go task performance, and ERP mean amplitudes for each component.

Variable	M(*SD)* or *N* (%)
Age (years)	6.26 (*1.5*)
**Biological sex**	
Male	13 (65%)
Female	7 (35%)
Average parental education	16.0^a^ (*1.9*)
Household income	$70,000–85,000 (range: $10,000–25,000 to $150,000–250,000)
CELF-5/CELF-P2	110.15 (*15.1*)
PTONI/TONI	109.85 (*22.9*)
Average screentime (minutes)	109.02 (*64.3*)
Go trials accuracy	95.33% (*3.8*)
No-Go trials accuracy	59.81% (*23.2*)
Go trials response time	411.71ms (*76.9*)
N1 Go amplitude	−21.43 μV (*8.1*)
N1 No-Go amplitude	−18.33 μV (*7.7*)
P2 Go amplitude	−14.25 μV (*7.3*)
P2 No-Go amplitude	−7.11 μV (*8.1*)
N2 Go amplitude	−21.93 μV (*10.9*)
N2 No-Go amplitude	−20.63 μV (*12.2*)
P3 Go amplitude	−4.88 μV (*6.8*)
P3 No-Go amplitude	1.99 μV (*7.7*)

a Average education level of standard college/bachelor’s degree.

## Data Availability

The raw data supporting the conclusions of this article will be made available by the authors, without undue reservation.

## Footnotes

1 The survey also included questions regarding content watched on devices, however there were no significant relationships with any variables and therefore we excluded content from our report.

## Appendix

Q1. Did your child spend time with any of the following devices today outside of school hours?

Please think across all non-school hours, including the morning, afternoon, and evening. Check all devices that your child spent any time with today.

- Computer (desktop or laptop) (1)
- Smartphone or Tablet (iPhone, Pixel, iPod Touch, iPad, KindleFire, GalaxyTab, etc.) (2)
- E-reader (Kindle, Nook, etc.) (3)
- Television (4)
- Video game device (Xbox, PlayStation, Nintendo DS, etc.) (5)
- Other device (please name or describe) (6):

Q2. How much time did your child spend on the computer (desktop or laptop)? [Dropdown box: hours 0–23, minutes 0–60]

Q3. How much time did your child spend on the smartphone or tablet)? [Dropdown box: hours 0–23, minutes 0–60]

Q4. How much time did your child spend on the e-reader? [Dropdown box: hours 0–23, minutes 0–60]

Q5. How much time did your child spend with the television? [Dropdown box: hours 0–23, minutes 0–60]

Q6. How much time did your child spend on the video game device (Xbox, PlayStation, Nintendo DS, etc.)? [Dropdown box: hours 0–23, minutes 0–60]

Q7. How much time did your child spend on the ?

[Dropdown box: hours 0–23, minutes 0–60]

Q8. Thinking across all those devices, which activities did your child do on any of those devices today? Check all that apply.

- Watch educational TV shows (OddSquad, Word World, WildKratts, etc.) (1)
- Watch entertainment TV/movies (SpongeBob Squarepants, Sofia the First, Disney movies, etc.) (2)
- Watch YouTube (3)
- Play educational apps (PBS Kids, ABC Mouse, Marble Math, Kodable, etc.) (4)
- Play entertainment apps (Angry Birds, Candy Crush, Toca Hair Salon, etc.) (5)
- Play online social games (Minecraft, Fortnight, etc.) (6)
- Read eBooks (7)
- Video Chat with others (FaceTime, Skype, etc.) (8)
- Use social media (TikTok, Facebook, Snapchat, etc.) (9)
- Send emails or text messages (gmail, iMessage, Facebook messenger) (10)
- Browse the internet (11)
- Other, please specify (12):
- Not sure. I did not closely monitor which activities my child chose (13).
