# Supplementary figures and images for: Children’s screentime is associated with reduced brain activation during an inhibitory control task: A pilot EEG study
Source: Front Cognit. Author manuscript; Available in PMC 2026 Jan 9. (PMC12782596; doi:10.3389/fcogn.2023.1018096)

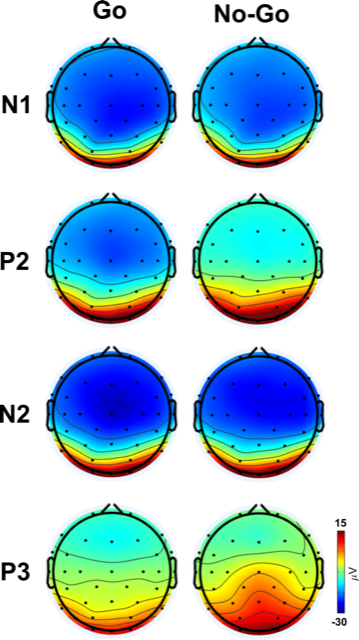

Supplement: Lewin et al 2023 - Supplementary Figure 2 — SUPPLEMENTARY FIGURE 2. Scalp topographies for each event-related brain potential (ERP) component averaged across the mean amplitude time window for Go (left) and No-Go (right) conditions. [file NIHMS2126586-supplement-Lewin_et_al_2023_-_Supplementary_Figure_2.tiff]

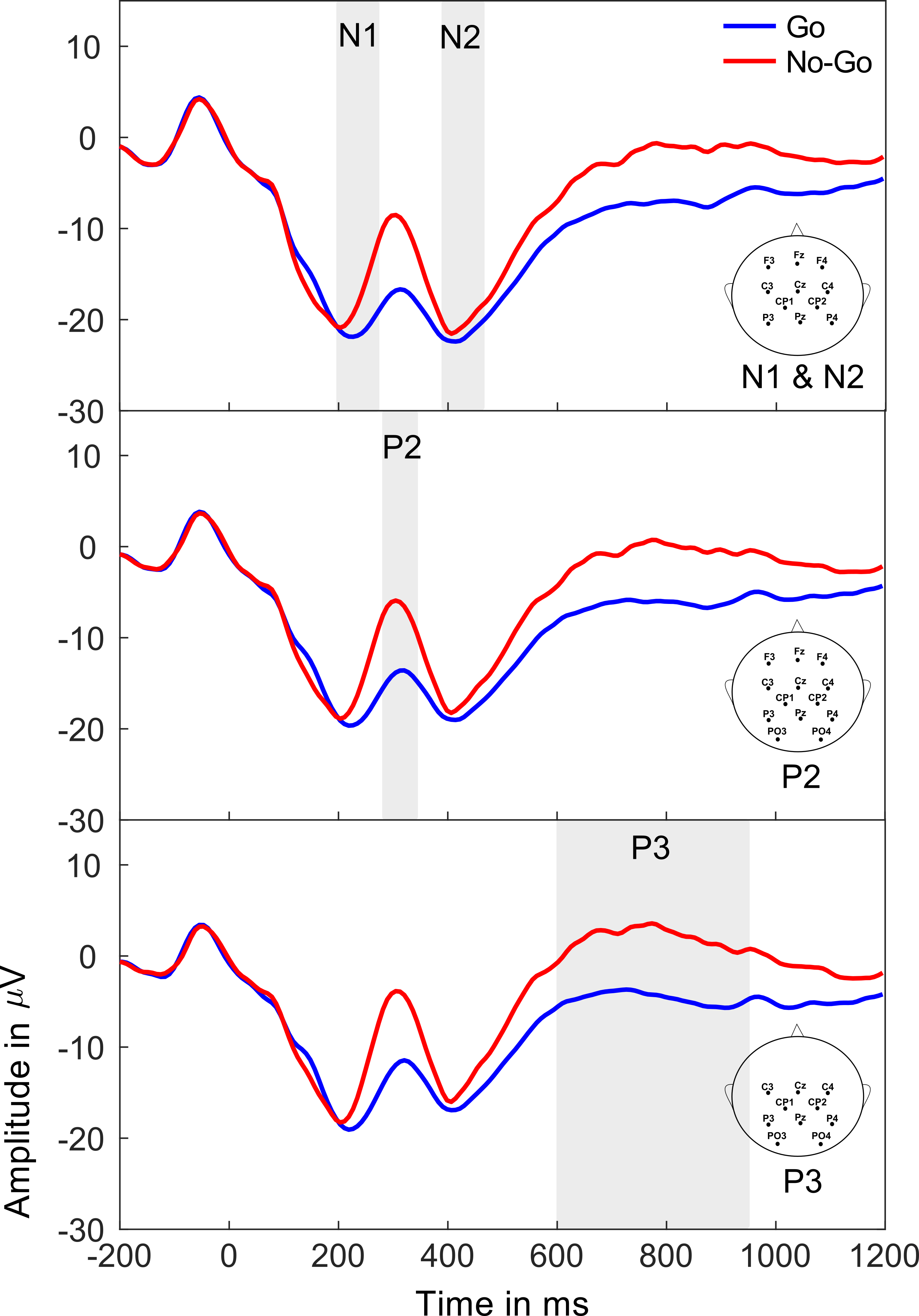

Supplement: Lewin et al 2023 - Supplementary Figure 1 — SUPPLEMENTARY FIGURE 1. Grand average event-related brain potential (ERP) waveforms for Go (blue) and No-Go (red) conditions averaged across the electrodes included in the region of interest (bottom right corner) for each component, N1 (top), N2 (top), P2 (middle) and P3 (bottom). Time windows in which mean amplitudes were extracted are shaded in gray. [file NIHMS2126586-supplement-Lewin_et_al_2023_-_Supplementary_Figure_1.tif]
